# An Improved Positioning Method for Two Base Stations in AIS

**DOI:** 10.3390/s18040991

**Published:** 2018-03-27

**Authors:** Yi Jiang, Jiani Wu, Shufang Zhang

**Affiliations:** Key Laboratory of Intelligent Waterway Transport Ministry of Transport, Information Science and Technology College, Dalian Maritime University, Dalian 116026, China; j_y@dlmu.edu.cn (Y.J.); sfzhang@dlmu.edu.cn (S.Z.)

**Keywords:** displacement correction position estimation (DCPE), accelerated motion, turning motion, automatic identification system (AIS)

## Abstract

Resilient position, navigation, and timing (PNT) data is indispensable information in the field of e-navigation. An automatic identification system (AIS) based ranging mode (R-Mode) is put forward to develop a terrestrial backup system in order to overcome the vulnerability of the global navigation satellite system (GNSS). In general, at least three base stations are required in the traditional R-Mode positioning method. However, the geometric distribution of existing base stations is not considered for positioning, as AIS is a communication system. In some cases, a vessel can only receive signals from two base stations. In this paper, an improved position estimation method based on displacement correction is therefore proposed to solve this problem. Compared with the prior displacement correction position estimation (DCPE) method, the proposed method can improve positioning accuracy effectively by adopting a more precise motion model for the vessel, including an accelerated motion and a turning motion model. Moreover, the motion model is employed adaptively to correct the displacement of the vessel. Finally, the proposed method is verified and the performance is analyzed and compared by simulation. This study can extend the application region of AIS R-Mode.

## 1. Introduction

The global navigation satellite system (GNSS) is the main navigation system in the international marine field at present. According to the e-Navigation strategy of the International Maritime Organization (IMO), resilient position, navigation, and timing (PNT) is the basic element. It is of great significance to having alternative terrestrial backup system to cope with a glitch or even a complete failure of GNSS [1]. Several navigation systems for maritime applications are developed, including the e-Loran system [2,3,4,5], inertial navigation system [6,7], terrain referenced navigation system [8], vessel traffic service and coastal surveillance system [9], etc. In the world-wide radio navigation plan (WWRNP) developed by the IMO and the International Association of Lighthouse Authorities (IALA), the ranging mode (R-Mode) based on the automatic identification system (AIS) as a ground-based alternative system to GNSS is listed explicitly. Therefore, many countries and scientists are concentrating on the new technologies of AIS R-Mode and demonstration systems [10]. In Europe, AIS R-Mode is a part of the Accessibility for Shipping, Efficiency Advantages and Sustainability (ACCSEAS) project. It is concentrated on the feasibility study of R-Mode using a combination of MF DGNSS, AIS, and eLoran, including the potential signals, geometry and signal strength, etc. [11,12]. Besides that, R-Mode in Baltic has been developed since October 2017 under the leadership of the German Aerospace Center (DLR) [13]. In China, the Maritime Safety Administration (MSA) and Dalian Maritime University have been investigating technologies for the AIS Autonomous Positioning System (AAPS), which achieves R-Mode positioning using AIS signals [14,15,16,17].

When the geometric distribution of AIS base stations is in good condition, a vessel can receive signals from no less than three base stations. Traditional positioning methods, such as Time of Arrival (TOA) or Time Difference of Arrival (TDOA), can be utilized to estimate vessel’s position. However, when the geometric distribution of base stations is poor, it is difficult to use traditional methods to determine positioning. For example, a vessel can only receive signals from two base stations. A displacement correction position estimation (DCPE) method was proposed to estimate positioning in the condition of two base stations as reference nodes for positioning in AIS [18]. The principle of DCPE is to calculate the displacement of the vessel for a period of time according to the heading and speed over ground provided by auxiliary sensors, such as compasses and log indicators, which have already equipped in vessels. However, the performance of the DCPE method is limited by the motion of vessels.

In this paper, an improved positioning method based on displacement correction is proposed to describe the movement more precisely for improving the positioning accuracy. The motion model of the prior DCPE method calculates displacement based on uniform rectilinear motion model, thus positioning errors can be larger sometimes (for example, when vessels are accelerating or turning a corner). In terms of acceleration, the proposed improved method calculates displacement by increasing estimated parameters in order to describe the motion more accurate. Consequently, displacement correction equations and positioning equations are both modified. Meanwhile, regarding turning motions, the improved DCPE method proposes a different model of displacement correction so that positioning errors can be reduced effectively. Besides that, the motion model is selected adaptively when the vessel’s position is estimated. Finally, the simulation results verify that the positioning accuracy of the proposed method is improved, especially when vessels are accelerating or turning.

The rest of this paper is organized as follows. Section 2 presents the principle of the DCPE method in AIS R-mode. In Section 3, the improved DCPE method is investigated. The improved positioning method during acceleration and turning are discussed, respectively. Section 4 gives the simulation scenario and results analysis and comparison between the proposed method and the prior DCPE method. Finally, some concluding remarks are put forth in Section 5.

## 2. Displacement Correction Position Estimation Method

**Figure 1:**
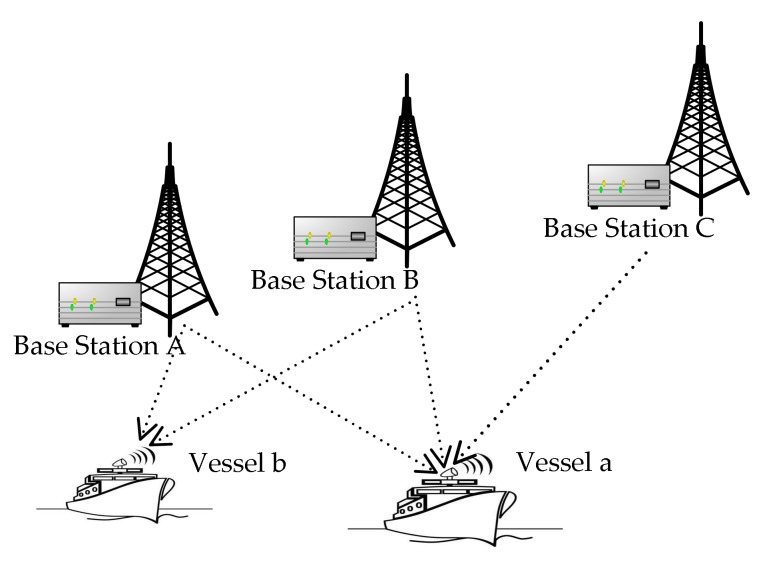
Scene of the automatic identification system (AIS) ranging mode (R-Mode) with two base stations.

The structure of the R-Mode positioning system based on AIS is illustrated in Figure 1. AIS base stations transmit the positioning signals periodically. The signals are then acquired, tracked, and demodulated by the vessel. Thus, transmission time between the vessel and different AIS base stations is measured. The product of transmission time and the speed of light is the distance. As the clock offset between the base stations and the vessel exists in the measured transmission time, the corresponding distances are the pseudoranges [19,20]. According to the demodulated messages from base stations, the precision position of base stations can be obtained. In general, the positioning method of AIS R-mode can be based on TOA or TDOA technology, if signals from at least three AIS base stations can be received, such as is the case for vessel a in Figure 1. If the vessel can only receive signals from two AIS base stations, such as in the case of vessel b in Figure 1, the DCPE method can be used to estimate the vessel’s position. The mathematical model and positioning principle of the DCPE method are described as follows.

**Figure 2:**
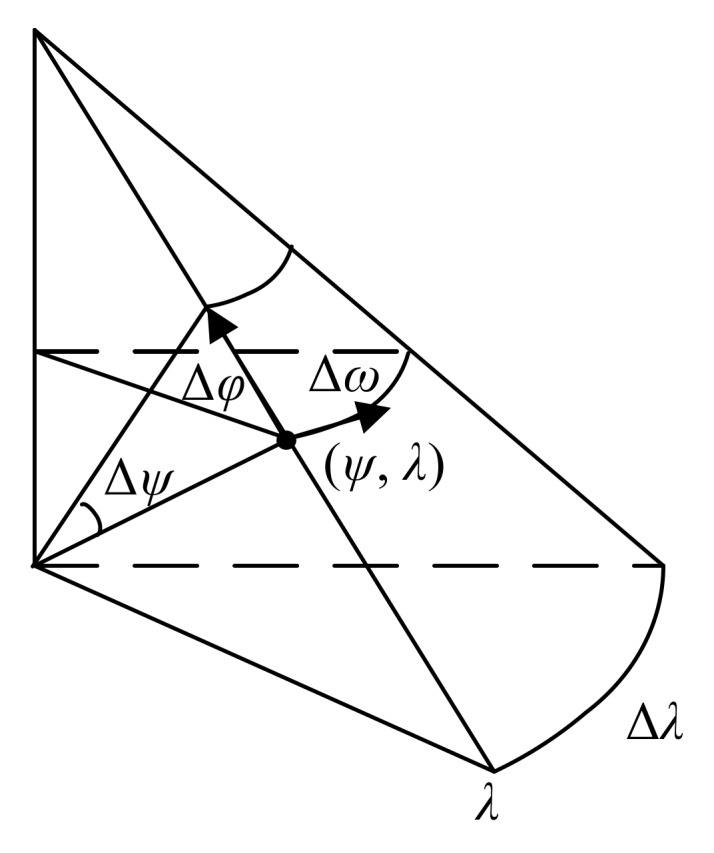
Schematic diagram of the relationship between (*ψ*, *λ*) and (Δ*φ*, Δω).

The DCPE method introduces the horizontal and vertical increments of the earth’s surface to estimate the position of vessel [21,22]. The geodetic coordinate is (*ψ*, λ). *ψ* and λ refer to latitude and longitude, respectively. On the surface of the earth, latitude variation Δ*ψ* corresponds to the horizontal distance Δ*φ* between the corresponding latitudes, whereas the vertical distance Δω equals Δλcos*φ* due to the shape of the earth. Δ*λ* is longitude variation. The relation between (*ψ*, λ) and (Δ*φ*, Δω) is shown in Figure 2.

At any time *k*, the geodetic coordinate of the vessel is indicated by (*ψ^k^*, *λ^k^*). Then (*ψ^k^*, *λ^k^*) can be converted into the horizontal and vertical coordinates (*φ^k^*, ω*^k^*) of the earth’s surface according to the Equation (1).

(1) $$\begin{array}{l} \varphi^{n}=\psi^{n}\\ \omega^{n}=\lambda^{n}\cos\varphi^{n} \end{array}\}$$

where the unit of the parameters is radian. Assuming that the vessel is with uniform rectilinear motion, the displacement (Δ*φ^k^*, Δω*^k^*) of the vessel after the time interval Δ*T* is given by

(2) $$\begin{array}{l} \Delta \varphi^{k}=v^{k}\Delta T\cos\alpha^{k}\\ \Delta \omega^{k}=v^{k}\Delta T\sin\alpha^{k} \end{array}\}$$

where *v^n^* is the speed over ground and α*^k^* is the heading of the vessel at time *k*, which can be both obtained according to real-time output of the auxiliary sensors on the vessel, such as compasses and log indicators.

The horizontal and vertical distance coordinates of the vessel after the time interval Δ*T* is denoted by (*φ^k^*^+1^, ω*^k^*^+1^), which can be calculated as Equation (3).

(3) $$\begin{array}{l} \varphi^{k+1}=\varphi^{k}+\Delta \varphi^{k}\\ \omega^{k+1}=\omega^{k}+\Delta \omega^{k} \end{array}\}$$

According to Equation (1), the geodetic coordinate (*ψ^k^*^+1^, *λ^k^*^+1^) of the vessel can be calculated by (*φ^k^*^+1^, ω*^k^*^+1^). 

Then, the distances between the vessel and the *i*th base station can be set as *L_i_*. At time *n*, the position equation is linearized using Taylor-series keeping only terms below the second order.

(4) $${\bar{L}}_{i}^{n}={\hat{L}}_{i}^{n}+\frac{\partial {\hat{L}}_{i}^{n}}{\partial \varphi }\delta \varphi +\frac{\partial {\hat{L}}_{i}^{n}}{\partial \omega }\delta \omega +c\delta t$$

where the subscript *i* = A, B referring to two AIS base stations; the superscript *n = k*, *k* + 1 has come to be different instants of time; ${\bar{L}}_{i}^{n}$ and ${\hat{L}}_{i}^{n}$ are the measured and estimated distance between the vessel and the base station, respectively; δ*φ* and δω indicate the correction of the horizontal and vertical coordinates respectively; δ*t* is the clock offset between the base station and the vessel, and *c* is the speed of light. The estimated distance ${\hat{L}}_{i}^{n}$ can be calculated by Equation (5).

(5) $${\hat{L}}_{i}^{n}=\cos^{-1}\left(\sin\varphi^{n}\sin\varphi_{i}+\cos\varphi^{n}\cos\varphi_{i}\cos\left(\lambda^{n}-\lambda_{i}\right)\right)$$

Finally, four positioning equations at *n = k*, *k* + 1 and *i* = A, B can be obtained according to measurements at two adjacent moments, which can be written in the form of the positioning matrix.

(6) $$\left[\begin{array}{c} {\bar{L}}_{A}^{k}-{\hat{L}}_{A}^{k}\\ {\bar{L}}_{B}^{k}-{\hat{L}}_{B}^{k}\\ {\bar{L}}_{A}^{k+1}-{\hat{L}}_{A}^{k+1}\\ {\bar{L}}_{B}^{k+1}-{\hat{L}}_{B}^{k+1} \end{array}\right]=\left[\begin{array}{ccc} \frac{\partial {\hat{L}}_{A}^{k}}{\partial \varphi } & \frac{\partial {\hat{L}}_{A}^{k}}{\partial \omega } & c\\ \frac{\partial {\hat{L}}_{B}^{k}}{\partial \varphi } & \frac{\partial {\hat{L}}_{B}^{k}}{\partial \omega } & c\\ \frac{\partial {\hat{L}}_{A}^{k+1}}{\partial \varphi } & \frac{\partial {\hat{L}}_{A}^{k+1}}{\partial \omega } & c\\ \frac{\partial {\hat{L}}_{B}^{k+1}}{\partial \varphi } & \frac{\partial {\hat{L}}_{B}^{k+1}}{\partial \omega } & c \end{array}\right]\left[\begin{array}{c} \delta \varphi \\ \delta \omega \\ \delta t \end{array}\right]$$

Therefore, the horizontal and vertical coordinates (*φ^k^*, ω*^k^*) of the vessel can be calculated after several iterations. According to Equation (1), the geodetic coordinate (*ψ^k^*, *λ^k^*) of the vessel at time *k* can be estimated.

## 3. Improved Displacement Correction Position Estimation Method

The DCPE method discussed above is based on a uniform rectilinear motion model. As the vessel is usually not uniform linear motion in reality, it will lead to large positioning error consequently in these situations. In view of the above problems, the improved method based on DCPE is investigated as below. First, the improved algorithm is discussed regarding acceleration and turning separately. Second, a different motion model is selected adaptively based on the estimated motion parameters during the vessel’s position estimation. Therefore, the displacement vector can be calculated more accurately, and positioning accuracy can be improved eventually.

### 3.1. Improved Method for Accelerated Motion

When the vessel is accelerating, the movement parameters are added during the calculation of the displacement in the improved method for improving the positioning accuracy. Assuming that the vessel makes accelerates uniformly, the initial position of the vessel is denoted by (*φ^k^*, ω*^k^*) at any time *k*. During the time interval Δ*T*, displacement vector of the vessel is $\Delta p^{k}=\left[\begin{array}{cc} \Delta \varphi^{k} & \Delta \omega^{k} \end{array}\right]$, which can be calculated as Equation (7).

(7) $$\Delta p^{k}=v^{k}\Delta T+\frac{1}{2}a^{k}{\left(\Delta T\right)}^{2}$$

where **v***^k^* and **a***^k^* are the speed over ground vector and the acceleration vector of the vessel at time *k* respectively. **v***^k^* can be divided into two components, $v_{H}^{k}$ and $v_{V}^{k}$. $v_{H}^{k}$ is the speed over ground in the direction of the horizontal, and $v_{V}^{k}$ is in the vertical direction.

(8) $$\begin{array}{c} v_{H}^{k}=\left|v^{k}\right|\cdot \cos\alpha^{k}\\ v_{V}^{k}=\left|v^{k}\right|\cdot \sin\alpha^{k} \end{array}\}$$

where *α^k^* is the heading angle in radian at time *k*. Similarly, the acceleration vector **a***^k^* can also be denoted like $a^{k}=[\begin{array}{cc} a_{H}^{k} & a_{V}^{k} \end{array}]$ as Equation (9).

(9) $$\begin{array}{c} a_{H}^{k}=\left|a^{k}\right|\cdot \cos\alpha^{k}\\ a_{V}^{k}=\left|a^{k}\right|\cdot \sin\alpha^{k} \end{array}\}$$

According to Equation (7), the displacement (Δ*φ^k^*, Δ*ω^k^*) after the time interval Δ*T* can be calculated by

(10) $$\begin{array}{l} \Delta \varphi^{k}=v_{H}^{k}\Delta T+\frac{1}{2}a_{H}^{k}{\left(\Delta T\right)}^{2}\\ \Delta \omega^{k}=v_{V}^{k}\Delta T+\frac{1}{2}a_{V}^{k}{\left(\Delta T\right)}^{2} \end{array}\}$$

Compared with Equation (2) in the DCPE method, the acceleration parameter is added in Equation (10) to calculate the displacement. Then the position of the vessel (*φ^k^*^+1^, *ω^k^*^+1^) can be obtained. Equation (3) is improved by

(11) $$\begin{array}{l} \varphi^{k+1}=\varphi^{k}+v_{H}^{k}\Delta T+\frac{1}{2}a_{H}^{k}{\left(\Delta T\right)}^{2}\\ \omega^{k+1}=\omega^{k}+v_{V}^{k}\Delta T+\frac{1}{2}a_{V}^{k}{\left(\Delta T\right)}^{2} \end{array}\}$$

The coefficients of first order items in positioning equation of Equation (4) can be expressed as

(12) $$\begin{array}{l} \frac{\partial {\hat{L}}_{i}^{n}}{\partial \varphi }=-\cos\beta_{i}^{n}\\ \frac{\partial {\hat{L}}_{i}^{n}}{\partial \omega }=-\sin\beta_{i}^{n} \end{array}\}$$

where $\beta_{i}^{n}$ is the azimuth of the *i*th base station relative to the vessel at time *n*, which can be calculated by

(13) $$\beta_{i}^{n}=\tan^{-1}\left(\frac{\cos\varphi_{i}\sin\left(\lambda_{i}-\lambda^{n}\right)}{\cos\varphi^{n}\sin\varphi_{i}-\sin\varphi^{n}\cos\varphi_{i}\cos\left(\lambda_{i}-\lambda^{n}\right)}\right)$$

Therefore, the geodetic coordinate (*ψ^k+^*^1^, *λ^k+^*^1^) of the vessel can be calculated using Equation (1) according to the proposed improved method in the condition of accelerated mode. Equation (6) can be simplified by

(14) b=A⋅x

where

(15) $$\begin{array}{ccc} x=\left[\begin{array}{c} \delta \varphi \\ \delta \omega \\ \delta t \end{array}\right], & b=\left[\begin{array}{c} {\bar{L}}_{A}^{k}-{\hat{L}}_{A}^{k}\\ {\bar{L}}_{B}^{k}-{\hat{L}}_{B}^{k}\\ {\bar{L}}_{A}^{k+1}-{\hat{L}}_{A}^{k+1}\\ {\bar{L}}_{B}^{k+1}-{\hat{L}}_{B}^{k+1} \end{array}\right], & A=\left[\begin{array}{ccc} \frac{\partial {\hat{L}}_{A}^{k}}{\partial \varphi } & \frac{\partial {\hat{L}}_{A}^{k}}{\partial \omega } & c\\ \frac{\partial {\hat{L}}_{B}^{k}}{\partial \varphi } & \frac{\partial {\hat{L}}_{B}^{k}}{\partial \omega } & c\\ \frac{\partial {\hat{L}}_{A}^{k+1}}{\partial \varphi } & \frac{\partial {\hat{L}}_{A}^{k+1}}{\partial \omega } & c\\ \frac{\partial {\hat{L}}_{B}^{k+1}}{\partial \varphi } & \frac{\partial {\hat{L}}_{B}^{k+1}}{\partial \omega } & c \end{array}\right] \end{array}$$

where the calculation of ${\hat{L}}_{A}^{k+1}$ and ${\hat{L}}_{B}^{k+1}$ is based on the improved method discussed above. And the correction vector **x** can be obtained by the least square method.

(16) $$x={\left(A^{T}A\right)}^{-1}A^{T}\cdot b$$

According to the Newton iterative method, the correction vector **x** is added to initial estimated position at each iteration to get the more accurate coordinate, which is also served as the initial estimated position of the next iteration. After a certain number of Newton iterations, the estimated position of the vessel would approach the real position at time *k* [23,24].

During the calculation of the displacement according to Equation (10), it should be noted that speed over ground vector **v** is provided by the auxiliary sensor on the vessel, such as a log indicator [25,26]. The acceleration vector **a** could be calculated by Equation (17).

(17) $$a^{k}=\frac{v^{k+1}-v^{k}}{\Delta T}$$

### 3.2. Improved Method for Turning Motion

When the vessel is turning, the position of the center of the turning is calculated in the improved method to assist in calculating the displacement for improving the positioning accuracy. The principle of the improved method for turning is described in detail below.

**Figure 3:**
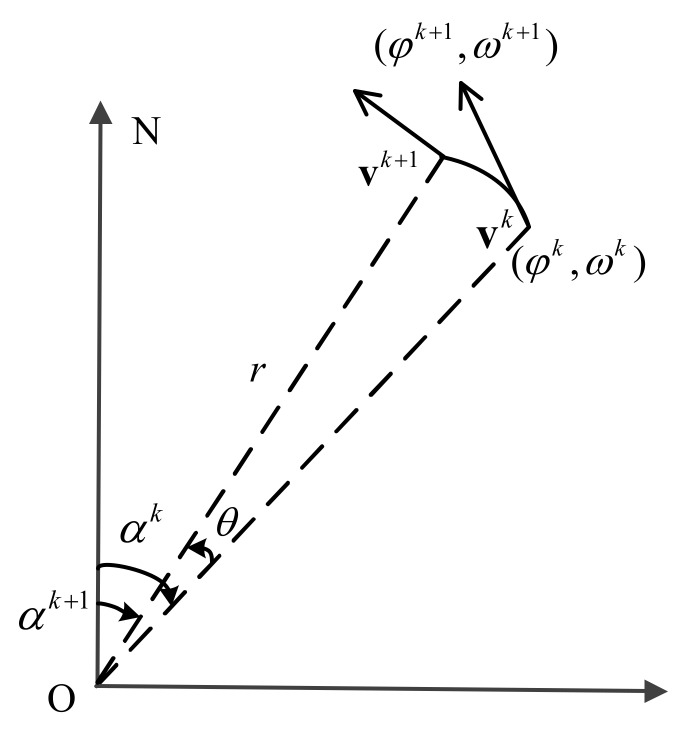
Geometrical model of turning motion.

Figure 3 shows the geometrical model of the vessel with turning motion. We assume that the direction of the speed over ground varies with time and the value of the speed over ground is constant at any moment when the vessel is turning. In Figure 3, O is the center and *r* is the radius of the turning corresponding to time *k*. During time interval Δ*T*, the trajectory of the vessel is in the shape of arc.

In Figure 3, the vessel is set to turn counterclockwise with the initial position (*φ^k^*, *ω^k^*) and the initial speed over ground **v***^k^* at time *k*. During time interval Δ*T*, the turning angle of the vessel is *θ*, if the counter clockwise direction is positive. 

First, the turning angle of the vessel *θ* is calculated. The speed over ground after time interval Δ*T* is **v***^k^*^+1^ during the turning. Similar to the speed over ground vector **v***^k^* at time *k*, it can also be divided into components in the horizontal and vertical direction. During the turning process, the relationship between **v***^k^* and **v***^k^*^+1^ is

(18) $$v^{k+1}=\left[\begin{array}{cc} v_{H}^{k+1} & v_{V}^{k+1} \end{array}\right]={\left(R\left(\theta \right)\left[\begin{array}{c} v_{H}^{k}\\ v_{V}^{k} \end{array}\right]\right)}^{T}$$

where *R*(*θ*) is called rotation matrix, defined by [27,28,29,30].

(19) $$R\left(\theta \right)=\left[\begin{array}{cc} \cos\theta & -\sin\theta \\ \sin\theta & \cos\theta \end{array}\right]$$

where *θ* equals to the product of angular velocity *ω^k^* and the time interval Δ*T* numerically.

Second, calculate the radius of the turning *r*. As the speed over ground vectors **v***^k^* and **v***^k^*^+1^ both can be measured by the auxiliary sensor on the vessel, *θ* can be calculated according to Equation (18). Dividing the time interval Δ*T*, the angular velocity *ω^k^* can be obtained then. The radius of the turning *r* can be calculated by

(20) $$r=\sqrt{{\left(v_{H}^{k}\right)}^{2}+{\left(v_{V}^{k}\right)}^{2}}/\omega^{k}$$

Third, the coordinate of the center of the turning O is (*O_H_*, *O_V_*), which can be calculated as Equation (21).

(21) $$\begin{array}{c} O_{H}=\varphi^{k}-r\cdot \sin(\alpha^{k})\\ O_{V}=\omega^{k}+r\cdot \cos(\alpha^{k}) \end{array}\}$$

Fourth, the position (*φ^k^*^+1^, *ω^k^*^+1^) of the vessel after the time interval Δ*T* during the turning can be calculated according to the center of the turning as Equation (22).

(22) $$\begin{array}{c} \varphi^{k+1}=O_{H}+r\cdot \cos(\alpha^{k}-\theta )\\ \omega^{k+1}=O_{V}-r\cdot \sin(\alpha^{k}-\theta ) \end{array}\}$$

Finally, the geodetic coordinate (*ψ^k+^*^1^, *λ^k+^*^1^) of the vessel can be calculated using Equation (1) according to the proposed improved method in the condition of turning mode. Then ${\hat{L}}_{A}^{k+1}$ and ${\hat{L}}_{B}^{k+1}$ in **b** defined in Equation (15) can be calculated. Then, the position of the vessel can be obtained using the least square method and the Newton iteration.

Therefore, the improved DCPE method estimates the vessel’s position (*φ^k+^*^1^, *ω^k+^*^1^) after the time interval Δ*T* using the above Equations (18)–(22), which is based on the geometric relationship in the model of turning motion. In contrast, the turning motion is converted into much a little rectilinear motion in the prior DCPE method, assuming that the trajectory of the vessel is approximated by a straight line in a very short time interval. So, it simply uses Equation (3) to estimate (*φ^k+^*^1^, *ω^k+^*^1^). Therefore, the proposed method is more accurate than the prior DCPE method.

### 3.3. Motion Model Adaptation Selection

It should be noted that the proposed improved DCPE method has different displacement update methods according to the kind of motion, including uniformly accelerated motion and turning motion. When the vessel’s position is estimated, we should judge what kind of motion the vessel is in at first. In the proposed improved method, the motion model is selected adaptively by calculation of the centripetal acceleration *a* according to

(23) $$a={\left(v_{H}^{k}\right)}^{2}+{\left(v_{V}^{k}\right)}^{2}/r$$

where $v_{H}^{k}$ and $v_{V}^{k}$ are the horizontal and the vertical component of the speed over ground, respectively. They can be calculated by Equation (8). 

According to the centripetal acceleration *a*, the corresponding improved DCPE method is adopted to estimate the vessel’s position. If centripetal acceleration *a* equals 0, the vessel is in rectilinear motion. We can utilize the improved DCPE method of accelerated motion to positioning. Otherwise, the vessel is in the turning motion. We can utilize the improved DCPE method of turning motion to achieve positioning.

**Figure 4:**
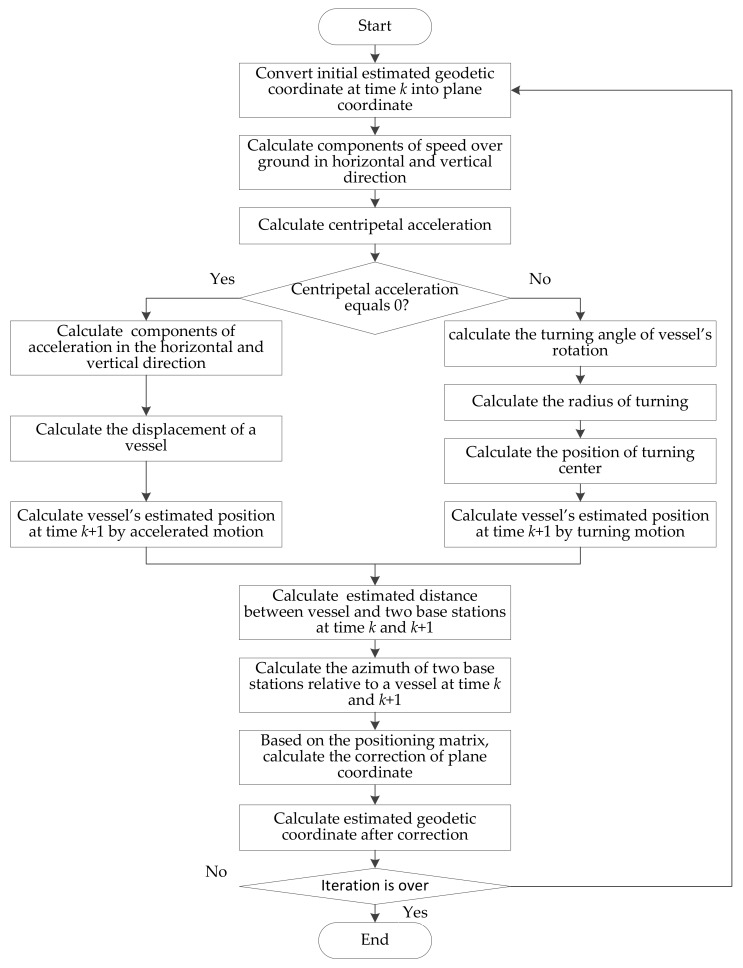
Flowchart of the improved displacement correction position estimation (DCPE) method.

Figure 4 is the flowchart of the proposed method.

First, the initial estimated position of the vessel $\left({\hat{\psi }}^{k},{\hat{\lambda }}^{k}\right)$ is converted into the horizontal and vertical coordinate $\left({\hat{\varphi }}^{k},{\hat{\omega }}^{k}\right)$ according to Equation (1). After that the motion model should be determined according to the centripetal acceleration *a*. The horizontal component $v_{H}^{k}$ and the vertical component $v_{V}^{k}$ of the speed over ground can be calculated as Equation (8). The speed over ground and the heading angle are provided by the auxiliary sensors on the vessel. Then Equation (23) is used to calculate *a*.

If the centripetal acceleration *a* equals zero, the accelerated motion mode is selected. The displacement of the vessel in the time interval Δ*T* can be obtained according to Equation (10). According to Equation (3) and Equation (1), the vessel’s estimated position $\left({\hat{\psi }}^{k+1},{\hat{\lambda }}^{k+1}\right)$ at time *k* + 1 can be obtained. Otherwise, if *a* does not equal zero, it turns to turning motion mode. Then according to Equation (18) to Equation (22), the vessel’s horizontal and vertical coordinate $\left({\hat{\varphi }}^{k+1},{\hat{\omega }}^{k+1}\right)$ after the time interval Δ*T* can be obtained. According to Equation (1), the vessel’s estimated position $\left({\hat{\psi }}^{k+1},{\hat{\lambda }}^{k+1}\right)$ at time *k* + 1 can be obtained.

Then the estimated distance and he estimated azimuth are calculated according to Equations (5) and (13), respectively. Based on the positioning matrix of Equation (6), the corrected values of horizontal and vertical direction are calculated using Equation (16). Then, vessel’s horizontal and vertical coordinate can be obtained by adding corrected values to initial estimated values. Finally, after several iterations, the position of the vessel (*ψ^k^*, *λ^k^*) can be obtained according to Equation (1).

## 4. Simulation Results and Analysis

### 4.1. Simulation Scenario

**Figure 5:**
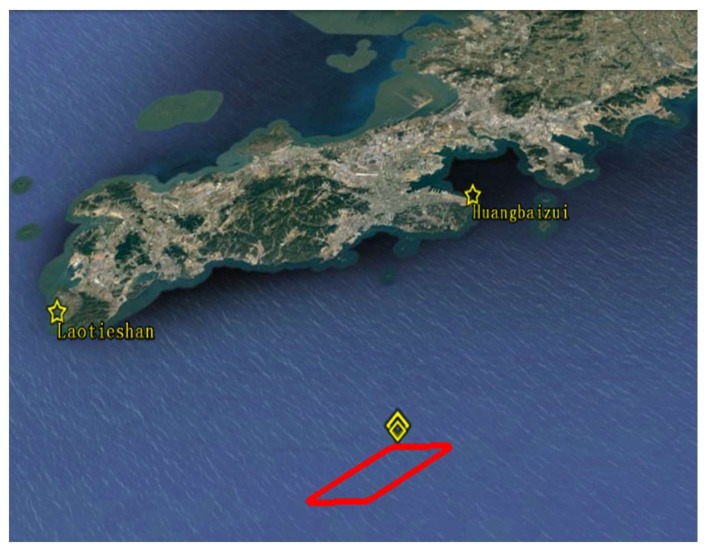
Simulation scenario.

The simulation scenario to verify the proposed method is shown in Figure 5. There are two real AIS base stations, marked by yellow five-pointed stars. Information on the AIS base stations is given in Table 1, including name, Maritime Mobile Service Identify (MMSI) code, and precise geodetic coordinates. MMSI is unique identification of the AIS base station or the vessel by IMO, which consists of nine bits of digital code [31,32].

In Figure 5, the initial position of the vessel is (38°34.414′ N, 121°38.187′ E), denoted by a yellow diamond. The vessel’s initial speed over ground is 5 m/s with an initial heading of 90°. First, the vessel moves in a uniformly accelerated rectilinear motion for 150 s with the acceleration of 0.2 m/s^2^. After that, the vessel moves in uniform rectilinear motion for 200 s. Then, the vessel moves in uniformly accelerated rectilinear motion for 150 s with the acceleration of −0.2 m/s^2^. After that, the vessel turns a corner with the centripetal acceleration of 0.156 m/s^2^, and the turning duration time is 50 s, corresponding to the upper right corner in Figure 5. Then, the vessel continually moves clockwise, and the motion of the vessel is similar to the above-mentioned process. Eventually the vessel’s trajectory is a red parallelogram, as shown in Figure 5. The destination is (38°34.421′ N, 121°38.193′ E), which is marked by a bigger yellow diamond. It can be seen in Figure 5 that the initial position and the destination could almost coincide.

### 4.2. Comparison and Analysis

#### 4.2.1. Horizontal and Vertical Errors

**Figure 6:**
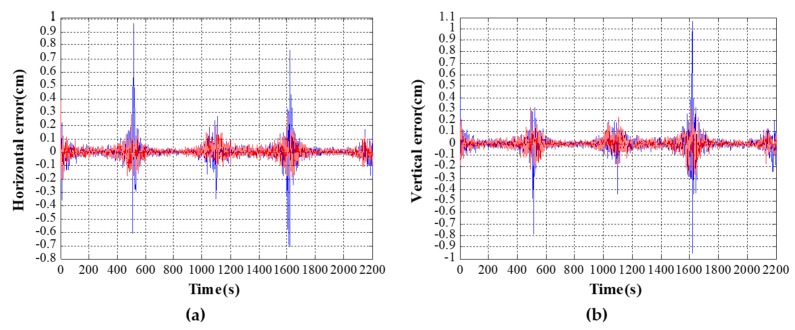
Deviation of positioning errors. (**a**) Horizontal errors; (**b**) Vertical errors.

The horizontal and vertical errors are compared between the DCPE method and the proposed method in the scenario described in Section 4.2 in Figure 6. The horizontal axis is time, while the vertical axis is the deviation of the positioning solution from the real position of the vessel per second. *δ_i_*(*t*) indicates the deviation, including the horizontal errors *δ*_1_(*t*) and vertical errors *δ*_2_(*t*). The mean of the deviation can be calculated by

(24) $$\mu_{i}=\sum_{n=1}^{N}\delta_{i}(t_{n})/N$$

where *t*_1_ indicates the starting moment and *N* is the total number of positioning. The standard deviation of errors is

(25) $$\sigma_{i}=\sqrt{{\sum_{n=1}^{N}\left(\delta_{i}(t_{n})-\mu_{i}\right)}^{2}/N}$$

In Figure 6, the deviation of the DCPE method is shown in the blue waveform and the deviation of the improved method is shown in the red waveform. From the simulation results, the mean of horizontal errors and vertical errors in the DCPE method are 0.0419 cm and 0.0752 cm, respectively. The standard deviation of horizontal errors in the DCPE method is 0.0696 cm, and the standard deviation of vertical errors is 0.0788 cm. In the meantime, the mean of horizontal and vertical errors in the improved method are −0.0135 cm and −0.0637 cm, respectively. The standard deviations of horizontal and vertical errors in the improved method are 0.0406 cm and 0.0461 cm, respectively. The detailed errors during each parts of movement are given in Table 2. *η* indicates the maximum error. It can be seen from Table 2 that positioning errors of the proposed method are smaller than those of the DCPE method. 

In the first straight trajectory of Figure 5, the vessel makes a uniformly accelerated rectilinear motion with acceleration of 0.2 m/s^2^ during the time period of 1~150 s. From the simulation results, the standard deviations of the horizontal and vertical errors using the DCPE method are 0.0560 cm and 0.0596 cm, respectively. While using the proposed method, the standard deviation of horizontal errors is 0.0514 cm, and the standard deviation of vertical errors is 0.0492 cm. Similarly, the vessel makes a uniformly accelerated rectilinear motion with acceleration of −0.2 m/s^2^ during the time period of 350~500 s. From the simulation results, the standard deviations of horizontal and vertical errors using the DCPE method are 0.0543 cm and 0.0605 cm, respectively. The standard deviations of horizontal and vertical errors using the proposed method are 0.0538 cm and 0.0489 cm.

From the blue waves of Figure 6a,b, it can be seen that there is larger deviation in two areas of each figure. Time of these two areas is 501~551 s and 1602~1652 s, which correspond to the upper right corner and the lower left corner of the trajectory in Figure 5. It can be seen that positioning error indicated by the blue waveform is larger in the above two areas, which means the performance of the proposed improved method is much better than the DCPE method during the turning. 

#### 4.2.2. Position Errors

**Figure 7:**
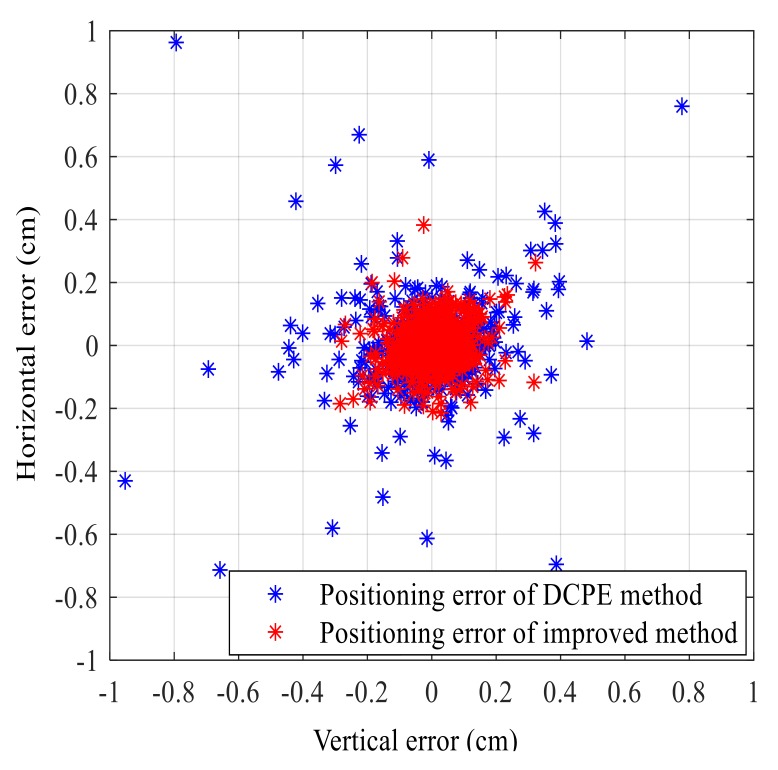
Comparison of positioning errors.

In addition, the position errors are illustrated in Figure 7 in the same simulation scenario described in Section 4.2. The blue and asterisks in Figure 7 show the dispersion of positioning errors using DCPE method and the improved method, respectively. As it can be seen from Figure 7, the dispersion of the improved method is more intensive than of the DCPE method. The standard deviation of positioning error using the DCPE method and using the improved method is 0.1052 cm and 0.0614 cm, respectively. The positioning error has been decreased for 41.6%. Thus, it verifies that the improved method has better positioning accuracy than the DCPE method. 

**Figure 8:**
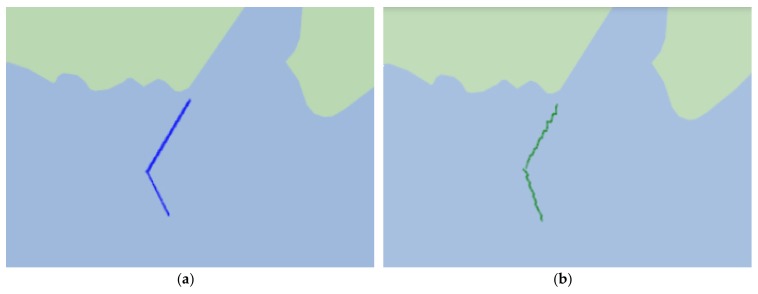
Trajectory of real motion. (**a**) Actual trajectory; (**b**) Estimated trajectory.

Besides that, we use the real motion of the vessel to verify the proposed method. The actual trajectory in Figure 8a is drawn according to the collected GNSS data of the vessel. The position is estimated using the proposed method. The speed over ground and the heading of the vessel are provided by a compass and a log indicator in the vessel, respectively. The trajectory based on the position estimation is drawn in Figure 8b. The mean of horizontal and vertical errors using the improved method are 1.2232 m and 1.1724 m, respectively. The standard deviations of horizontal and vertical errors in the improved method are 7.9451 m and 8.6367 m, respectively. The positioning errors are mainly due to the errors including the speed over ground and the heading errors.

#### 4.2.3. Errors Analysis

In order to analyze the reason of the performance improvement of the proposed method more clearly, the details of positioning errors and azimuth angles between the vessel and the base station are given. Simulation results using the DCPE method and the proposed method in the scenario described in Section 4.2 are given in Table 3 and Table 4, respectively. *β_A_* and *β_B_* are the azimuth angles Laotieshan and Huangbaizui in Figure 5. Data in Table 3 and Table 4 corresponds to a part of the first turning process in Figure 5 during the 505th to 521st second. Horizontal error and vertical error represent positioning errors in the horizontal and vertical direction when the vessel is in turning motion.

It can be seen from the Table 3 that, initially, *β_A_* and *β_B_* change in the same trend. At the third point, *β_A_* gradually increases, while *β_B_* continually decreases. In this process, *β_A_* and *β_B_* change in the opposite trend, which corresponds to data in blue font in the first two columns. After 15 points, *β_A_* and *β_B_* change in the same trend again. 

Simulation data shows that the larger positioning errors, marked in red in the last two columns in Table 3, all appear in the area where *β_A_* and *β_B_* change in the opposite trend, which corresponding to the area of the upper right corner or the lower left corner in Figure 5. This directly leads to a large value of (**A***^T^***A**)^−1^**A***^T^* in the Equation (16), which results in a larger Δ*x* in each iteration.

Generally speaking, in the Newton iteration process, when the longitude and latitude gradually converge, there will be a certain degree of fluctuation [33,34]. The value of Δ*x* becomes smaller with the gradual convergence of the positioning in the iteration. When the Newton iteration converges to specified accuracy, the value of Δ*x* is very small. In the region of above two corners, the value of Δ*x* is still large, which leads to the visible fluctuation. Even at the end of the iteration, there is still a large deviation between positioning results and the actual trajectory. Therefore, there is a sudden increase of positioning errors of latitude and longitude in the blue simulation waveform in Figure 6.

Simulation results using the proposed method are shown in Table 4. By comparing the data in Table 3 and Table 4, it can be observed that errors of the proposed method are greatly reduced from the 507th second to the 519th second. All the above-mentioned elements have been marked in red font in the corresponding position of Table 3 and Table 4.

It can be seen that the situation of sudden increase of positioning errors in turning motion is greatly improved after adopting the improved method. As shown in the red waveform in Figure 6, the positioning error at the turning almost does not increase. Compared with the DCPE method, the improved method has the obvious effect of improvement.

The reason is that the improved method is calculated on the basis of the geometric principle of the curved arc, and the corrected value Δ*x* is smaller after the convergence. While in the DCPE method, the curved arc is approximated as a straight line, which leads to a larger corrected value Δ*x*.

During the area of the upper right corner in Figure 5, corresponding to the time period of 501~551 s in Figure 6, the standard deviation of horizontal and vertical errors using the DCPE method are 0.2727 cm and 0.2401 cm, respectively. The standard deviations of horizontal and vertical errors using the proposed method are 0.0811 cm and 0.0851 cm, respectively. 

Similarly, in the area of lower left corner in Figure 5, corresponding to the time period of 1602~1652 s in Figure 6, the standard deviations of horizontal and vertical errors using the DCPE method are 0.0898 cm and 0.1103 cm, respectively. The standard deviations of horizontal and vertical errors using the proposed method are 0.0419 cm and 0.0538 cm, respectively.

In summary, according to the above simulation results and analysis, the improved method can effectively reduce positioning errors during uniform acceleration and turning and consequently reduce positioning errors in the overall motion.

## 5. Conclusions

The R-Mode positioning system based on AIS, which is advocated by IMO, is a terrestrial backup navigation system to overcome the vulnerability of GNSS. The proposed method based on DCPE is used to estimate the vessel’s position when signals from only two AIS base stations can be received. In such conditions, the traditional positioning method cannot be used. The prior DCPE method estimates the vessel’s position based on the model of uniform rectilinear motion, and therefore the position errors may be large in some situations (for instance, when the vessel is accelerating or turning). The proposed method in this paper improves the calculation of the displacement based on the mathematical models of accelerating and turning motions. In addition, the motion model is selected adaptively when the vessel’s position is estimated. The performance of the DCPE method and the improved method is compared in the same simulation scenario. The reason for the sudden increase of positioning errors in some area using the DCPE method is analyzed. According to the simulation results, the positioning errors of the proposed method decrease 41.6%. Even in the condition of real motion of the vessel with errors of the auxiliary sensors, the positioning accuracy is acceptable.

## Figures and Tables

**Table 1:** Information on AIS base stations.

Name	MMSI	Latitude	Longitude
Laotieshan	4,131,101	$38{}^{\circ}43.6420^{\prime }$ N	$121{}^{\circ}08.1330^{\prime }$ E
Huangbaizui	4,131,104	$38{}^{\circ}54.2850^{\prime }$ N	$121{}^{\circ}42.9500^{\prime }$ E

**Table 2:** Detailed errors during each parts of movement.

Method	Error	1~500 s	501~550 s	551~1050 s	1051~1100 s	1101~1600 s	1601~1650 s	1651~2150 s	2151~2200 s
DCPE Method	*η*_1_	0.3826	0.9627	0.1457	0.2402	0.2710	0.7601	0.1382	0.1689
	*η*_2_	0.3934	0.3165	0.1948	0.1868	0.2575	1.0614	0.2291	0.1959
	*μ*_1_	−0.0010	0.0276	−0.0007	0.0120	0.0009	0.0249	−0.0010	−0.0262
	*μ*_2_	0.0014	−0.0724	0.0019	−0.0195	−0.0025	0.0524	0.0003	−0.0175
	*σ*_1_	0.0444	0.2673	0.0251	0.1024	0.0459	0.2624	0.0271	0.0674
	*σ*_2_	0.0445	0.2358	0.0329	0.1107	0.0534	0.3429	0.0353	0.0808
Improved Method	*η*_1_	0.2180	0.2043	0.1048	0.1996	0.1462	0.2633	0.1057	0.0766
	*η*_2_	0.3174	0.1674	0.1805	0.1200	0.2280	0.3222	0.1816	0.0780
	*μ*_1_	−0.00001	−0.0019	0.0001	0.0023	−0.0008	0.0128	−0.0003	−0.0035
	*μ*_2_	0.0004	0.0177	0.0016	0.0018	−0.0006	0.0229	−0.0007	0.0001
	*σ*_1_	0.0426	0.0800	0.0244	0.0494	0.0420	0.0952	0.0259	0.0390
	*σ*_2_	0.0442	0.0834	0.0302	0.0612	0.0466	0.1236	0.0347	0.0415

**Table 3:** Azimuth angles and positioning errors in the turning using DCPE method.

No.	$\beta_{A}^{k}$	$\beta_{B}^{k}$	Horizontal Error	Vertical Error
1	5.009371978	6.116692350	0.136755441	−0.033688209
2	5.009364103	6.116582698	−0.044890233	0.178701572
3	5.009360177	6.116480498	0.133527424	−0.354018592
4	5.009360530	6.116386250	−0.613144241	−0.014801477
5	5.009364834	6.116299943	0.038769839	−0.400999096
6	5.009373314	6.116221335	−0.481755011	−0.151736104
7	5.009385773	6.116150846	−0.083722842	−0.476183031
8	5.009402483	6.116088651	−0.233052817	0.274446059
9	5.009422998	6.116034472	0.458114784	−0.422002996
10	5.009447685	6.115988481	0.589510173	−0.008883503
11	5.009476324	6.115950728	0.115658450	−0.192622662
12	5.009508806	6.115921259	0.962727494	−0.793874836
13	5.009545403	6.115900175	−0.075081556	−0.693991153
14	5.009585874	6.115887465	−0.175334603	−0.333361476
15	5.009630160	6.115883378	0.669634066	−0.225495902
16	5.009678183	6.115887176	0.202885538	−0.298708880
17	5.009730051	6.115899711	0.259243149	−0.218549897

**Table 4:** Azimuth angles and positioning errors in the turning of improved algorithm.

No.	$\beta_{A}^{k}$	$\beta_{B}^{k}$	Horizontal Error	Vertical Error
1	5.009371995	6.116692369	0.052833868	0.038729669
2	5.009364061	6.116582636	0.072796110	−0.032886895
3	5.009360236	6.116480577	0.059390886	−0.007025953
4	5.009360511	6.116386303	0.036391146	0.029988286
5	5.009364878	6.116299822	−0.137737964	0.088876636
6	5.009373319	6.116221349	0.006360655	0.085991809
7	5.009385838	6.116150860	0.015130796	0.111074183
8	5.009402406	6.116088433	−0.190604914	−0.026010386
9	5.009423040	6.116034233	−0.068020249	0.087595125
10	5.009447679	6.115988256	−0.033166051	0.009970006
11	5.009476329	6.115950615	0.097515659	0.020200008
12	5.009508939	6.115921190	0.003789785	0.041866405
13	5.009545475	6.115900117	0.017615842	0.063760294
14	5.009585895	6.115887434	0.056106653	0.006849625
15	5.009630184	6.115883145	0.102804730	0.105033462
16	5.009678249	6.115887169	0.038196622	0.049230991
17	5.009730064	6.115899593	0.051263916	−0.011125038
